# Light-Emitting-Diode-Induced Fluorescence from Organic Dyes for Application in Excitation–Emission Fluorescence Spectroscopy for Food System Analysis

**DOI:** 10.3390/foods13091329

**Published:** 2024-04-26

**Authors:** Veselin Vladev, Mariya Brazkova, Stefan Bozhkov, Galena Angelova, Denica Blazheva, Stefka Minkova, Krastena Nikolova, Tinko Eftimov

**Affiliations:** 1 Department of Mathematics, Physics and Information Technologies, Faculty of Economics, University of Food Technologies, 26 Maritsa Blvd., 4002 Plovdiv, Bulgaria; v_vladev@uft-plovdiv.bg (V.V.); sbozhkov@uft-plovdiv.bg (S.B.); kr.nikolova@abv.bg (K.N.); 2 Central Laboratory of Applied Physics, Bulgarian Academy of Sciences, 61 Sankt Peterburg Blvd., 4002 Plovdiv, Bulgaria; tinko.eftimov@uqo.ca; 3 Department of Biotechnology, Technological Faculty, University of Food Technologies, 26 Maritsa Blvd., 4002 Plovdiv, Bulgaria; g_angelova@uft-plovdiv.bg; 4 Department of Microbiology, Technological Faculty, University of Food Technologies, 26 Maritza Blvd., 4002 Plovdiv, Bulgaria; d_blazheva@uft-plovdiv.bg; 5 Department of Physics and Biophysics, Medical University—Varna, 84 Tzar Osvoboditel Blvd., 9000 Varna, Bulgaria; stefka.minkova@mu-varna.bg; 6 Centre de Recherche en Photonique, Université du Québec en Outaouais, 101 rue Saint-Jean-Bosco, Gatineau, QC J8Y 3G5, Canada

**Keywords:** LED-induced fluorescence, organic dyes, EVOO, polycyclic aromatic hydrocarbons, broadband light source, optical fibers, Czerny–Turner monochromator, diffuse reflector, barium sulfate, polyvinyl alcohol

## Abstract

An experimental study is presented on the possibility of using the fluorescence from organic dyes as a broadband light source together with a monochromator for applications in excitation–emission matrix (EEM) fluorescence spectroscopy. A high-power single-chip light-emitting diode (LED) was chosen as an excitation source with a central output wavelength at 365 nm to excite a fluorescent solution of Coumarin 1 dye dissolved in ethanol. Two excitation configurations were investigated: direct excitation from the LED and excitation through an optical-fiber-coupled LED. A Czerny–Turner monochromator with a diffraction grating was used for the spectral tuning of the fluorescence. A simple method was investigated for increasing the efficiency of the excitation as well as the fluorescence signal collection by using a diffuse reflector composed of barium sulfate (BaSO_4_) and polyvinyl alcohol (PVA). As research objects, extra-virgin olive oil (EVOO), Coumarin 6 dye, and Perylene, a polycyclic aromatic hydrocarbon (PAH), were used. The results showed that the light-emitting-diode-induced fluorescence was sufficient to cover the losses on the optical path to the monochromator output, where a detectable signal could be obtained. The obtained results reveal the practical possibility of applying the fluorescence from dyes as a light source for food system analysis by EEM fluorescence spectroscopy.

## 1. Introduction

Fluorescence spectroscopy is a rapid analytical technique, characterized by simplicity and cost-effectiveness, for the characterization of molecules or events into fluorescing samples. It is successfully applied in many fields such as forensic, agricultural, and pharmaceutical science, bioscience, and medical diagnostics, as well as food safety and quality control [1,2,3,4,5,6]. A typical fluorescence measurement involves irradiating the sample with constant monochromatic light, usually set to the maximum of a given fluorophore absorption curve, and detecting the resulting fluorescence signal. This approach is typically used when analyzing a sample that contains a single fluorophore. The studied samples usually are complex systems composed of multiple fluorophores whose absorption and emission spectra may be overlapped or not. In such samples, changing the excitation wavelength causes the emitted fluorescence spectrum to change as well.

One of the methods used to analyze complex fluorescent samples is EEM fluorescence spectroscopy. EEM fluorescence spectroscopy is a highly sensitive and non-destructive 3D steady-state fluorescence technique that is a variation of standard fluorescence spectroscopy [7]. It allows for the simultaneous visualization of all the fluorophores of a complex sample in a single three-dimensional plot. In this technique, the sample is excited with a monochromatic light at several different wavelengths and the emitted fluorescence is acquired at each excitation. With the excitation–emission data, 3D or contour plots can be obtained, which could serve as a fluorescence fingerprint.

In the context of human nutrition, food represents a complex system, containing a variety of proteins, carbohydrates, fats, vitamins, minerals, and other components. In order to achieve a pleasant appearance and flavor and to expand the expiration date, often, artificial additives are used in the manufacturing process. Despite the quality control in the food manufacturing sector, there are cases of contamination or even adulteration. Food adulteration refers to the deliberate deception or misrepresentation of food products for economic gain. Many nutrients contained in the food matrix are naturally fluorescent; thus, the fluorescence spectrum of the sample could be the sum of the individual spectra of the fluorophores presented in it.

On the other hand, environmental pollution in combination with the methods of food processing are the main sources for the contamination of foods with PAHs. These organic compounds contain two or more fused aromatic rings and due to their persistence, are widespread in the environment [8]. The development of methods for continuously monitoring the presence of PAHs in foods is necessary mainly because they can affect human health after dietary exposure and cause immunosuppressive, carcinogenetic, and mutagenetic effects [9,10,11]. The reference method for the determination of PAHs in food samples [12] combines thin-layer chromatography with subsequent UV spectrophotometry. The main disadvantages here are related to the high solvent volumes and low sensitivity. Also, the methods for the extraction, identification, and quantification of PAHs in food samples are time consuming and challenging.

In this case, EEM fluorescence spectroscopy in combination with statistical methods was successfully applied for the analysis of food samples [13,14]. This method has been applied for the analysis of a variety of food products such as edible oils [15,16], honey [17], water [18], and edible insects [19], as well as toxins that contaminate foods [20,21].

The light source is a core component of any spectroscopic technique. Typically, for EEM fluorescence spectroscopy, powerful lamps are used as a broadband light source in continuous or pulsed mode in combination with a monochromator [22,23,24,25,26,27,28,29]. The main disadvantages of these lamps are that they are bulky, expensive, and have a high energy consumption.

As an alternative, laser diodes (LDs) and LEDs could be used to obtain fluorescence spectra [30,31,32,33,34,35,36,37,38,39]. A characteristic feature of the application of LDs and LEDs as fluorescence-inducing sources is that no spectral selection mechanism is used, namely, a monochromator. Instead, spectral selection is achieved by using individual LDs and LEDs emitting at a specific wavelength. In principle, LDs are characterized by a lower beam divergence of the radiation emitted by them compared to LEDs. This allows for easier focusing of their radiation on the sample by means of optics, which induce strong fluorescence. LDs with output wavelengths in the visible spectrum down to 405 nm, which emit an optical power in the range of a few mW to a few W, are commercially available at an affordable price.

Regarding the spectral width of their output, of the order of 1 nm, LDs can be classified as linear light sources. Thus, their spectral parameters correspond very well with the characteristic features of EEM fluorescence spectroscopy. Unfortunately, the range of available LDs wavelengths is limited.

Unlike LDs, LEDs with a larger wavelength variety, covering the visible and ultra-violet regions down to 250 nm, are commercially available at an affordable price. The variety of optical powers, beam divergences, and spectral widths is significant. It can be summarized that the beam divergences are greater than those of LDs, typically 120° for a single-chip LED. The spectral width of their output on the order of 20 nm classifies them as a band light source. The optical powers on the order of several hundred mW are emitted by LEDs with wavelengths above 370 nm. For LEDs emitting below 370 nm, the optical power decreases dramatically, with the price for the more powerful ones increasing significantly.

The laser-induced fluorescence from highly efficient organic dyes can be used for the creation of broadband light sources for applications in light spectroscopy, for example, all-fiber miniature light sources based on organic fluorescent dyes [40,41,42,43], in which fiber-optic ferrules and capillary tubes are used as the basic constructions. The possibility of coupling them not only to conventional optical fibers but also to hollow-core and photonic-crystal fibers combined with fiber-optic micro-optics has been demonstrated. In this way, their possible practical applications in the field of photonics could be expanded. The stability and suitable mechanical properties of the ferrule by achieving laser generation in an all-fiber liquid dye laser has also been demonstrated [44,45]. The potential of the laser-induced fluorescence from organic dyes to be used as a light source for EEM fluorescence spectroscopy by combining them with a monochromator has also been demonstrated [46].

In the present study, we demonstrate that the LED-induced fluorescence from an organic dye can be used as a light source for EEM fluorescence spectroscopy. To the best of our knowledge, no such approach has been reported in the scientific literature to date.

## 2. Materials and Methods

### 2.1. Fluorescent Medium

Two organic fluorescent dyes, Coumarin 1 and Rhodamine 6G (Merck KGaA, Darmstadt, Germany), were used in the experiments, which have quantum yields of 0.73 and 0.95, respectively. Coumarin 1 was dissolved in 99.9% ethanol with a dye concentration of 4.59 × 10^−3^ M and Rhodamine 6G was dissolved in 99.9% glycerol with a dye concentration of 1.19 × 10^−4^ M. An analytical balance was used to prepare the fluorescent media (AS 82/220.R2 PLUS, RADWAG, Radom, Poland).

An EVOO, a Coumarin 6 dye (Merck KGaA, Darmstadt, Germany), and the PAH Perylene (Merck KgaA, Darmstadt, Germany) were used as research objects. The EVOO was purchased from a local supplier and was used with no further treatments. Coumarin 6 was dissolved in 99.9% ethanol with a dye concentration of 8.35 × 10^−4^ M and had a quantum yield of 0.78. Perylene 99.5% was dissolved in 99.9% cyclohexane (Honeywell Riedel-de Haën, Charlotte, NC, USA) with a concentration of 1.33 × 10^−3^ M and had a quantum yield of 0.94.

### 2.2. Exciting and Receiving Components

An LED and a laser were used to induce fluorescence from the active media, which were powered by a linear laboratory power supply (AX-3003L-3, Axiomet, Kraków, Poland).

A surface-mounted high-power LED was used (GD35-X-365-DL, Roithner Lasertechnik GmbH, Wien, Austria) with a peak wavelength of 365–375 nm, output optical power of 500–700 mW, and was mounted on a heatsink. The LED was composed of a single die emitter with a factory-installed quartz lens, as it emits through a beam angle of 120°.

The laser used was a continuous-wave diode-pumped solid-state (DPSS) Nd:YAG laser emitting at 532 nm, having a maximum output power of 5 mW.

**Figure 1:**
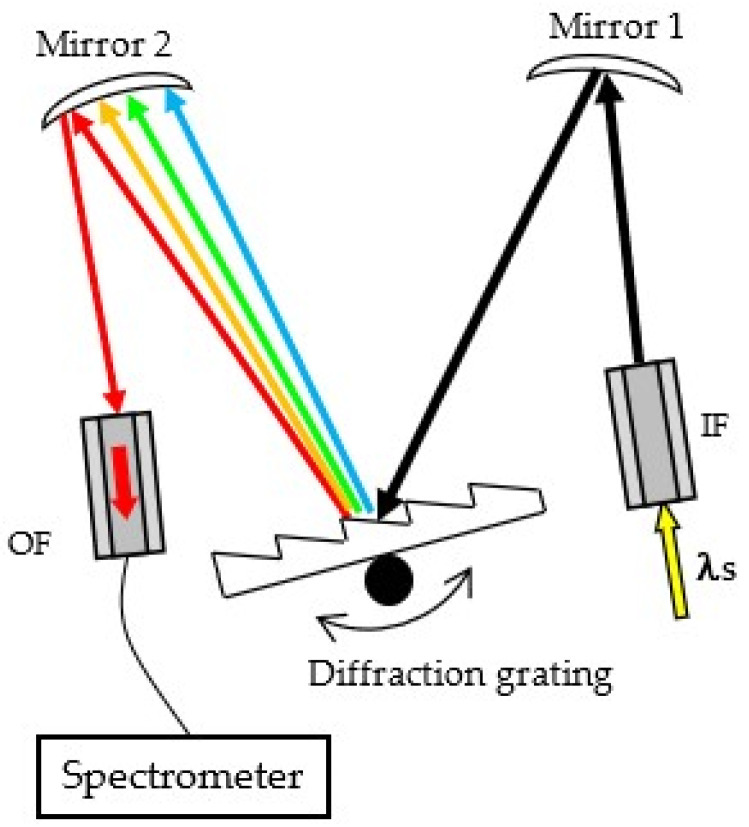
Schematic view of the monochromator used.

**Figure 2:**
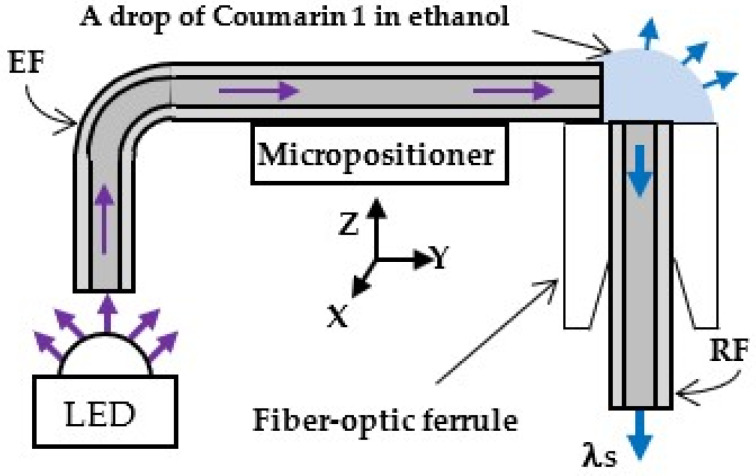
Experimental set-up of the dye solution excitation scheme through an optical fiber. In the case of LED excitation, the solution was Coumarin 1 in ethanol. In the case of laser excitation, the solution was Rhodamine 6G in glycerol.

**Figure 3:**
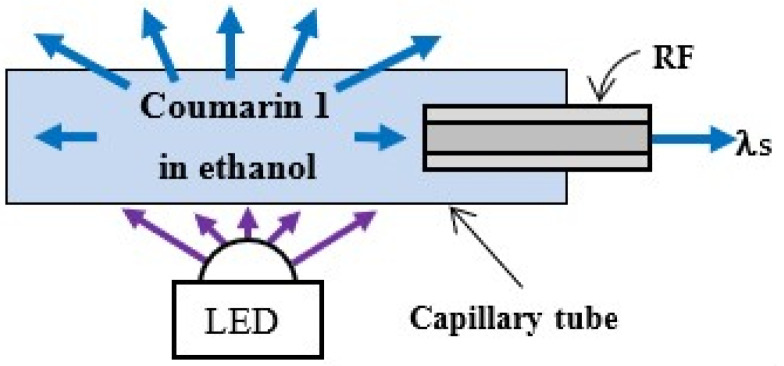
Experimental set-up of the direct LED excitation of Coumarin 1 in ethanol.

The fibers from Figure 1, Figure 2 and Figure 3 were multimode fused silica step-index optical fibers (FG105UCA, Thorlabs Inc., New Jersey, NY, USA) with 105 μm/125 μm core/cladding diameters and numerical aperture NA = 0.22. There are optical fibers with larger cores, but we have deliberately chosen to limit ourselves to the largest core diameter with 125 μm cladding. In this way, we deliver as much light as possible while ensuring the high resolution of the output of the monochromator.

### 2.3. Experimental Set-Up

For the experiment, a Czerny–Turner monochromator with a diffraction grating was assembled, which is presented schematically in Figure 1. No special slits were used at either the input or the output of the monochromator. Optical fibers with 105 μm core diameters were placed at the entrance and at the exit of the monochromator, the cores of which serve as slits. The input optical fiber (IF) and the output optical fiber (OF) were mounted in zirconia ferrules and were polished with an optical fiber polishing machine (FibrMet, Buehler Ltd., Lake Bluff, IL, USA) using diamond polishing papers with a decreasing grain size, namely, 30 μm, 9 μm, 3 μm, and 1 μm. The prepared fibers were mounted in fiber ferrule clamps (Thorlabs Inc., New Jersey, NY, USA).

A plane-ruled reflection grating placed on a diffraction grating mount was used, with maximum efficiency at 300 nm (Newport Corp., Irvine, CA, USA). The grating was rotated manually by using a rotational micropositioner stage (M-481-A, Newport Corp., Irvine, CA, USA) which allows for precise manual adjustments of 0.1° by means of a screw. UV-enhanced aluminum concave mirrors mounted in kinematic mirror mounts were used, which, together with all the other components, were mounted on an aluminum optical breadboard (Thorlabs Inc., New Jersey, NY, USA).

To observe the output spectra, a fiber-optic spectrometer was used (AvaSpec-HERO, Avantes, Apeldoom, The Netherlands) with a wavelength range of 250–626 nm, slit size of 25 μm, and resolution of 0.85 nm. The spectrometer was connected with a solarization-resistant fiber patch cable with a core diameter of 105 μm (Thorlabs Inc., New Jersey, NY, USA).

Two configuration schemes for fluorescence induction were investigated: an illumination of the active medium through an excitation optical fiber (EF) connected to an LED and a direct excitation through an LED, presented in Figure 2 and Figure 3, respectively.

In the scheme in Figure 2, the radiation from an LED or a laser excites the active medium in front of the facet of a receiving optical fiber (RF). The RF was placed in a zirconia fiber-optic ferrule for stability whereby the optical axes of EF and RF are at a 90° angle. The resulting fluorescence signal λ_S_ was waveguided from the RF to the IF of the monochromator. The EF was fixed to a micropositioner having three linear displacements and a two-axis tilt platform (ULTRAlign 561D and 561-TILT-LH, Newport Corp., Irvine, CA, USA). The position of the fiber tip was precisely controlled with the micropositioner.

In the scheme in Figure 3, the active medium was illuminated directly by the LED. The fluorescence solution was placed in a capillary tube with a RF was placed inside to receive the fluorescence λ_S_ and the tube was placed directly on the LED lens. The capillary tube was made of borosilicate glass with an inner diameter of approximately 343 μm and a wall thickness of approximately 64 μm. The tube was manually drawn from a larger-diameter tube using a butane gas torch as a heating source.

**Figure 4:**
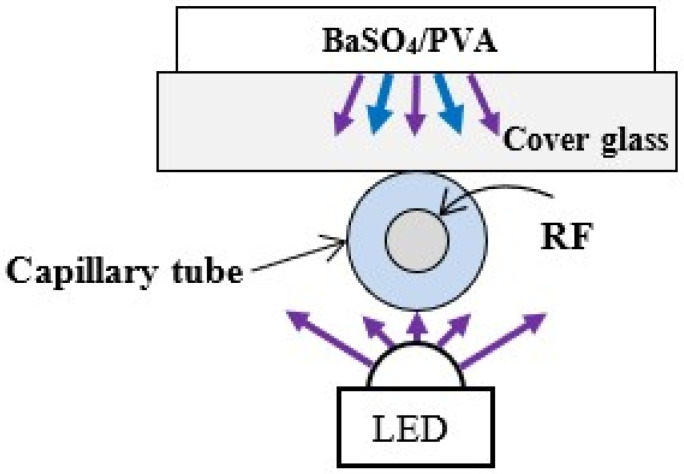
Experimental set-up of the direct LED excitation configuration with a BaSO_4_/PVA diffuse reflector on a cover glass.

To demonstrate the possibility of increasing the LED excitation as well as accepting the fluorescence received by the fiber, we used a diffuse reflective layer composed of BaSO_4_ dissolved in PVA [47,48] applied to a cover glass. The PVA was dissolved in distilled water at 90 °C and stirred manually until a homogeneous solution was obtained. Then, the BaSO_4_ powder was added, after which the mixture was stirred manually until thickened. The solution was dropped onto a cover glass, which was left on a hot plate at 30 °C until the water evaporated and a solid layer was formed. The prepared diffuse reflector was placed on the capillary tube, as shown in Figure 4.

## 3. Results

**Figure 5:**
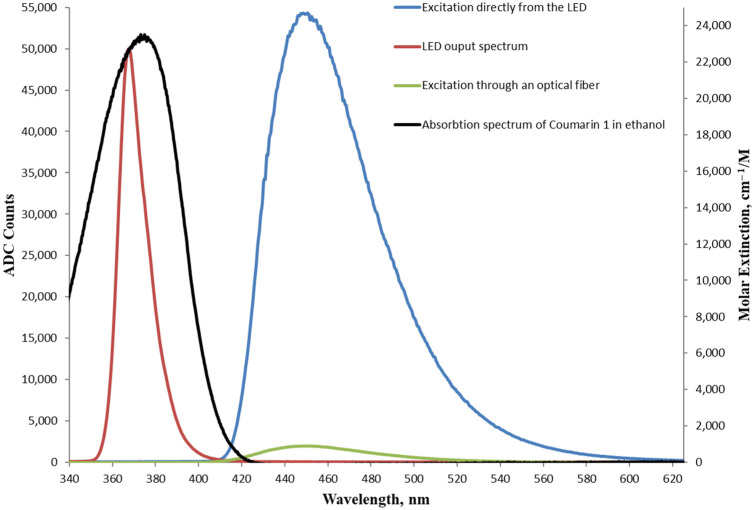
Fluorescence spectra of Coumarin 1 in ethanol upon excitation at 365 nm directly by an LED, as well as through an optical fiber. The absorption spectrum of Coumarin 1 in ethanol is also presented [49].

Figure 5 shows the obtained fluorescence spectra of Coumarin 1, excited, respectively, through an optical-fiber-coupled LED and direct excitation by an LED. The corresponding excitation set-up schemes are presented in Figure 2 and Figure 3, respectively. The spectra were obtained at the spectrometer’s minimum integration time of 5.22 ms. Also, the figure shows the output spectrum of the LED at 367 nm obtained through an EF, with the EF and spectrometer fiber spaced apart to prevent detector saturation, which occurs at 65130 ADC counts.

The LED was fed with a forward current value of 90 mA, where the full width at the half maximum (FWHM) of 15.6 nm of the LED spectrum was measured. The LED output wavelength was close to the absorption maximum of Coumarin 1 at 373 nm [49], which, combined with the good overlap of the LED spectrum with the dye absorption curve, resulted in very efficient excitation.

The resulting fluorescence spectrum of the transverse excitation through an optical-fiber-coupled LED was centered at 450 nm and had an FWHM of 59.6 nm. As can be seen, the intensity is sufficient and can be easily detected by the spectrometer, but will not be sufficient to cover the losses on the way to the output of the monochromator despite the high power of the LED. We attribute this to the characteristic feature of LEDs of their emission area being greater compared to that of LDs.

**Figure 6:**
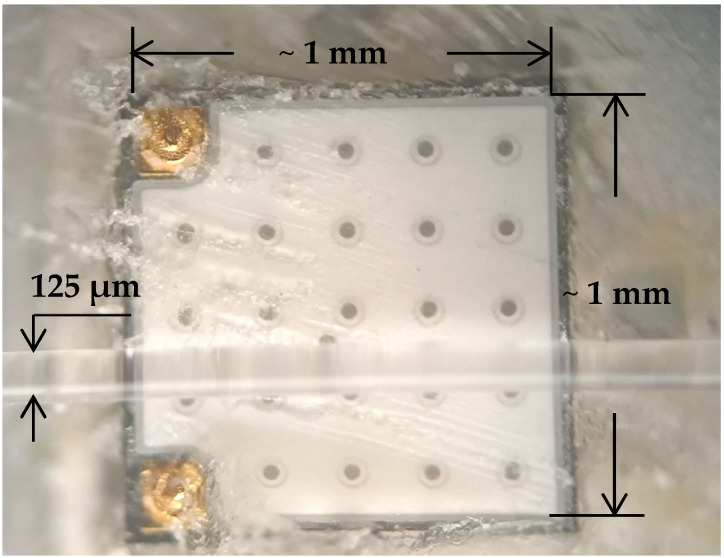
An optical microscope image of the LED chip with an optical fiber on it with dimensions for comparison.

Figure 6 shows the die emitter of the same LED type and with the lens removed, as well as an optical fiber placed on top with a cladding diameter of 125 μm for comparison. The picture was taken through an optical microscope (Olympus, Tokyo, Japan), and the dimensions of the LED emitting surface were determined to be approximately 1 mm × 1 mm. This means that a significant part of the output radiation of the LED was not accepted by the optical fiber; therefore, the intensity of the excitation radiation from the fiber was too low and insufficient to cause significant fluorescence. For this reason, we resorted to the scheme of Figure 3, where the receiving fiber, together with the capillary tube, were positioned in such a way as to obtain a maximally intense fluorescent signal.

The resulting fluorescence spectrum of the direct excitation by an LED is shown in Figure 5 and had a central wavelength of 450 nm and an FWHM of 58 nm, again with the RF and the spectrometer fiber slightly decoupled to prevent detector saturation. Clearly, with this excitation configuration, the intensity of the excitation radiation was significant and the fluorescence signal easily saturated the detector even at a minimal integration time value.

**Figure 7:**
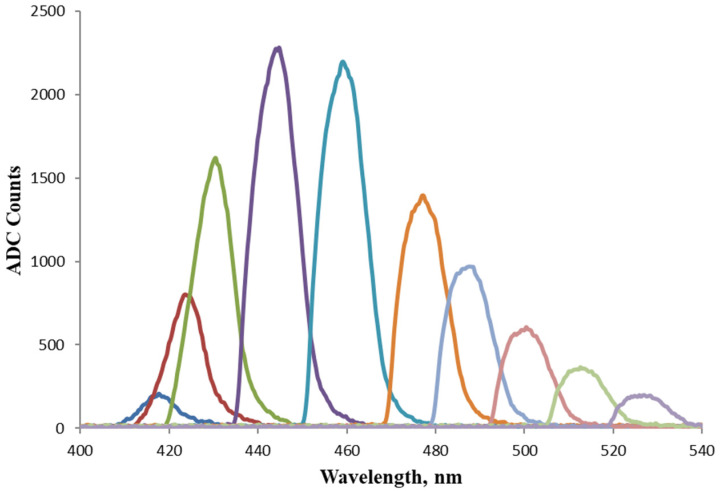
Output spectra from the monochromator after wavelength tuning the fluorescence signal from Coumarin 1 in ethanol directly excited by a LED.

Figure 7 shows a series of spectra from the monochromator output after tuning according to the scheme presented in Figure 3. The peak of the maximum intensity had an FWHM of 11.6 nm. The difference in the signal intensity before and after the monochromator is significant due to energy losses at the fiber connectors, losses associated with the focusing zone in front of the monochromator OF facet, and also from diffraction from the grating itself.

**Figure 8:**
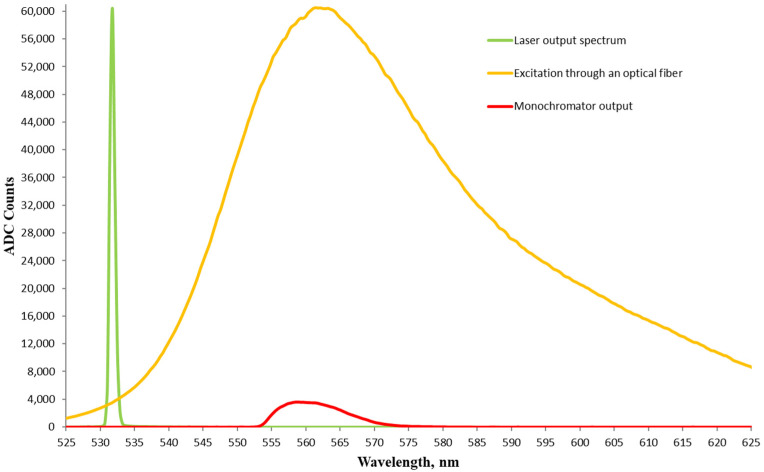
Fluorescence spectra of Rhodamine 6G in glycerol excited with a fiber-optic coupled DPSS Nd:YAG laser. The fluorescence spectra are at the input and at the output of the monochromator. Also shown is the excitation laser peak at 532 nm.

For comparison with an excitation source other than an LED, the scheme in Figure 2 was used, in which the difference was that the LED was replaced by a DPSS Nd:YAG laser and the dye was replaced by Rhodamine 6G. Figure 8 shows the laser spectrum with a peak at 531.7 nm and an FWHM of 0.74 nm, the fluorescence spectrum of Rhodamine 6G before the monochromator with an FWHM of 39.6 nm as well as after the monochromator with an FWHM of 11.7 nm centered at 558.8 nm. Again, the spectra were acquired at an integration time of 5.22 ms.

The spectra from the laser source and the fluorescence before the monochromator were at slightly spaced connectors to keep the maximum intensities below the saturation level. It can be seen that the radiation intensities after the monochromator with laser excitation through an optical fiber and with direct LED excitation were similar, despite the higher optical power of the LED. This is primarily due to the smaller emitter area of the laser, resulting in a higher radiation density, and easier coupling to the optical fiber.

**Figure 9:**
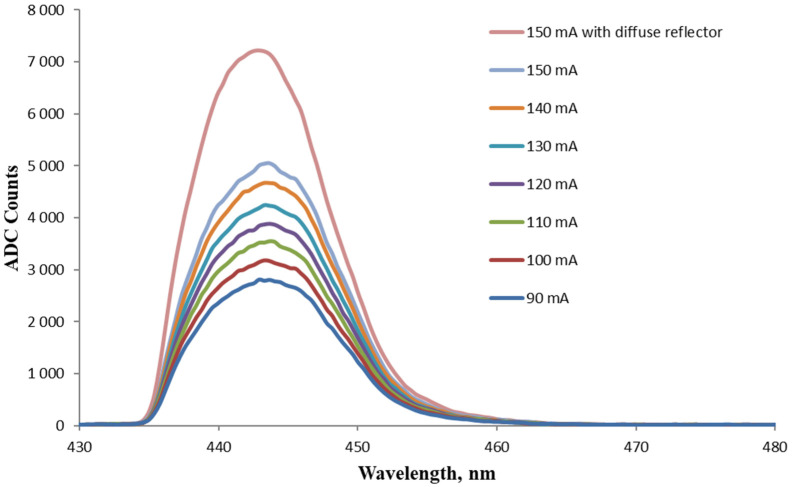
Output spectra from the monochromator using direct LED excitation of Coumarin 1 in ethanol at the LED current values between 90 mA and 150 mA as well as when using BaSO_4_/PVA reflector.

Due to the high divergence of the excitation radiation from the LED and the capture of only part of the fluorescent signal, which was emitted in all directions, the efficiency of the whole process was lowered. In an attempt to increase the useful fluorescent signal, the LED was driven with a forward current ranging from 90 mA to its typical working value of 150 mA with a 10 mA increment. The resulting spectra after the monochromator are presented in Figure 9, where an increase in the peak value of the output intensity is observed with uniform steps corresponding to the driving current of the LED.

To limit the spread in space of a part of the excitation radiation and also of the fluorescence signal, we used a low-cost and easy-to-manufacture optical element in the form of a diffuse reflector. As a reflective substance, BaSO_4_ powder (having high reflectivity across the UV-VIS-NIR range) and a PVA binder (with low absorbance in UV-VIS range) was used. The prepared BaSO_4_/PVA reflector was used as shown in Figure 4, and the effect of using it at an LED forward current of 150 mA is shown in Figure 9. As can be seen, even such a simply made reflector gives a tangible positive effect, leading to an increase in the intensity of the useful signal.

**Figure 10:**
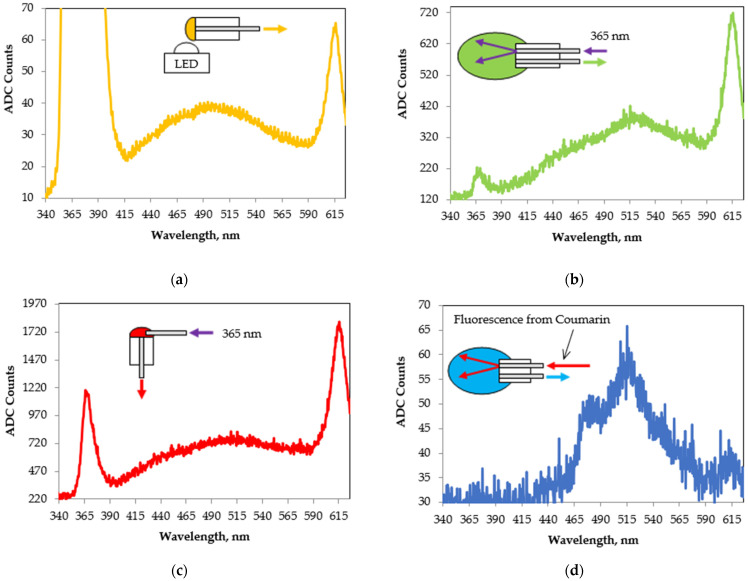
Fluorescence spectra from EVOO with (**a**) transverse excitation by direct LED illumination; (**b**) longitudinal excitation through an optical-fiber-coupled LED; (**c**) transverse excitation through an optical-fiber-coupled LED; (**d**) longitudinal excitation through an optical fiber with the fluorescence of Coumarin 1.

Figure 10 presents four fluorescence spectra from the same EVOO obtained with different sample excitation schemes and with different spectral sources. The insets on each of the figures present the schemes used for the excitation and reception of the signal.

In the case of Figure 10a, a drop of EVOO was placed on the facet of an optical fiber mounted on a single-hole zirconia ferrule, and then positioned over the LED for illumination. The intense peak around 370 nm is unabsorbed radiation from the LED. We assume that the peak around 615 nm is associated with the fluorescence of pigments from the chlorophyll group. Similar results were presented by other research groups previously [50,51].

In the case of Figure 10b, a scheme with longitudinal excitation from an optical-fiber-coupled LED was tested. A borosilicate glass ferrule was used with an outer diameter of 2.3 mm and a length of 10.4 mm with two parallel openings along its length located around its center, both with inside diameters of 125 μm. Two optical fibers with core diameters of 105 μm were placed in them, one for the excitation radiation and the other for receiving the fluorescence. The two optical fibers were fixed with an optical glue and their faces were polished on a polishing machine. The ferrule was placed in a tube that was filled with the EVOO sample. The small peak around 370 nm was a reflection at the EVOO/air interface. Visual observation of the irradiated zone shows strong fluorescence of a red color, which we assume was from the characteristic strong peaks of chlorophyll and pheophytin around 670 nm, which is reported in the literature [52]. We failed to detect this peak due to the spectral limitation of the used spectrometer at 626 nm.

In the case of Figure 10c, we used a transverse excitation through an optical-fiber-coupled LED. The excitation radiation from the LED was directed by means of an optical fiber and a micropositioner to the front of the RF, as shown in Figure 2, where a drop of the EVOO sample is located. The peak around 370 nm was also from a reflection at the EVOO/air interface. It can be seen that in all three cases of the LED excitation, the obtained spectra were similar.

In the case of Figure 10d, longitudinal excitation from an optical-fiber-coupled LED was used again, but this time, the excitation radiation was the fluorescence spectrum of Coumarin 1 induced by using the scheme of Figure 4. The peak at 515 nm in Figure 10d and also that in Figure 10b are associated with the presence of vitamin E. The peak at 480 nm and the weak peak around 445 nm in Figure 10d are associated with the presence of oxidative products [53,54,55,56]. The obtained spectrum at an integration time of 1 s was rather weakly intense. For this reason, we did not try excitation with radiation from the monochromator because we were unable to detect the more intense fluorescence from EVOO after 626 nm, which is commonly reported in the literature [52].

We therefore decided to use the highly efficient fluorescent dye Coumarin 6, which emits its peak within the spectral range of our spectrometer.

**Figure 11:**
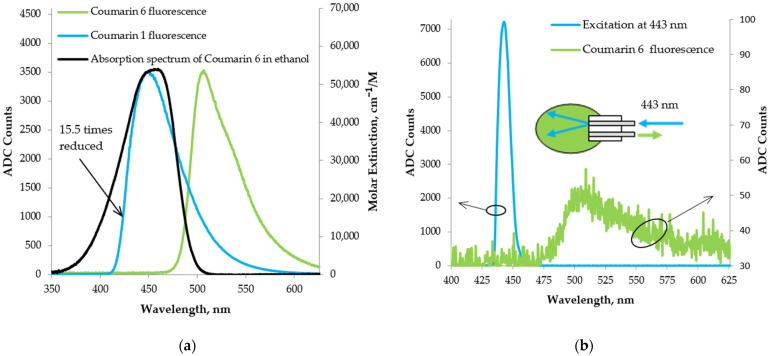
Fluorescence spectra from Coumarin 6 in ethanol obtained with longitudinal excitation through an optical fiber with (**a**) the fluorescence from Coumarin 1 in ethanol; (**b**) a 443 nm output from the monochromator.

Figure 11a shows the Coumarin 6 spectrum obtained under longitudinal excitation through an optical fiber with fluorescence from Coumarin 1, a significant portion of which overlaps the absorption spectrum of Coumarin 6 [57]. As can be seen from Figure 11a, the obtained fluorescence at an integration time of 1 s is clearly pronounced.

In Figure 11b, the obtained spectrum from Coumarin 6 is shown with longitudinal excitation through an optical fiber with radiation from the monochromator at 443 nm. The excitation spectrum falls entirely into the absorption spectrum of Coumarin 6, close to its maximum at 457 nm [57]. In the same figure, the inset shows the scheme used to capture the fluorescence of Coumarin 6. The obtained spectra show that the signal was detectable, thereby demonstrating the ability to detect a specific fluorophore. However, the fluorescence obtained was quite weak as it was observed for an integration time of 1 s. This requires further improvements to be made to the LED-induced fluorescence scheme to increase the intensity of the output radiation from the monochromator.

**Figure 12:**
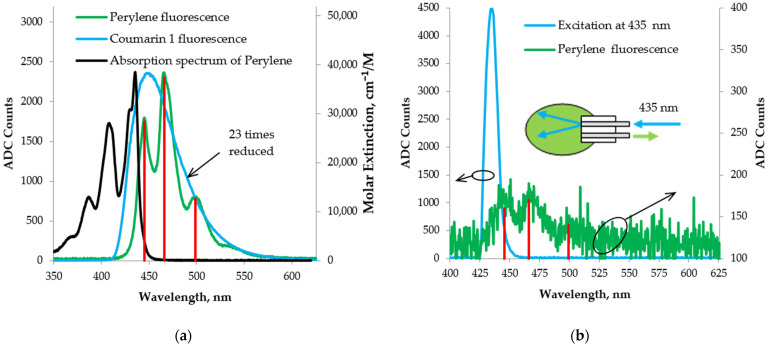
Fluorescence spectra from Perylene in cyclohexane obtained with longitudinal excitation through an optical fiber with (**a**) the fluorescence from Coumarin 1 in ethanol; (**b**) a 435 nm output from the monochromator.

Figure 12a shows the spectrum of Perylene obtained under longitudinal excitation through an optical fiber with fluorescence from Coumarin 1, which partially overlaps the absorption spectrum of Perylene [58]. The resulting fluorescence signal was clearly pronounced despite the slight overlap between the absorption and excitation spectra. We believe that this is because of the sufficiently intense excitation fluorescence radiation. In Figure 12a, the red lines mark the three characteristic Perylene fluorescence peaks, respectively, at 445.5 nm, 466 nm, and 500 nm.

In the case of Figure 12b, the Perylene was longitudinally excited through an optical fiber with radiation from the monochromator at 435 nm, which coincided very well with the maximum absorbance peak from the Perylene curve. Despite the large losses to the output of the monochromator and the fact that the excitation radiation at 435 nm was not maximally intense, we still obtain a detectable signal. Moreover, the characteristic fluorescence peaks of Perylene are clearly distinguishable. The peaks at 445.5 nm and 466 nm are clearly distinguished and we may speculate about the presence of the peak at 500 nm, all marked with red lines in Figure 12b.

## 4. Discussion

The obtained results show that the observation of fluorescence by excitation of the dye solution from a fiber-coupled LED does not yield sufficient power despite the high optical power of the LED and the factory-installed optics. This is due to the large divergence of the excitation light and the large emitting surface of the LED chip compared to the diameter of 105 μm of the EF core. In this way, the EF receives a small fraction of the LED radiation, resulting in a lower energy density in the excitation region of the fluorescent medium.

From the comparison made with laser excitation through an optical fiber, it can be seen that similar results comparable to those with direct LED excitation are easily achieved. It should be noted that this is the case when using an excitation laser source of significantly lower power, at a few mW at most. This opens up the possibility of using commercially available high-power laser diodes or DPSS lasers, which would significantly increase the intensity of the output fluorescence signal. As previously demonstrated by us [40,41,42,43,44,45], the use of fiber micro-optics further increases the flexibility of such fiber-coupled laser-excited broadband sources. In this way, potential opportunities for new research in this direction are revealed.

Based on the experimental results, we can conclude that in the case of LED-induced fluorescence, a configuration with direct LED excitation is more suitable compared to the excitation through a fiber-coupled LED. We demonstrate the two main approaches to increase the efficiency of LED-induced fluorescence. One way to increase the excitation efficiency is to use the LED’s maximum capability to emit light without being damaged, namely, by increasing the forward current. The other is to use an optical element, such as the demonstrated BaSO_4_/PVA diffuse reflector, to concentrate the excitation radiation onto the dye solution and direct more fluorescence to the RF. As can be seen from the presented results, the second approach with a diffuse reflector has potential for development and future experimentation in this direction. For example, increasing the concentration of BaSO_4_ in the composition of the reflector and optimizing its shape will potentially lead to the better collection of the dye fluorescence. The use of more than one LED for fluorescence excitation, and using dyes with high quantum efficiencies, could potentially lead to a significant increase in the fluorescence intensity.

The resulting monochromator spectrum with an FWHM of the order of 11 nm is comparable to that of the LED excitation source, which is, in principle, used itself as an excitation source for EEM fluorescence spectroscopy. It should be noted that the obtained fluorescence spectra were produced using a highly sensitive fiber-optic spectrometer with a 25 μm input slit illuminated by a 105 μm core optical fiber. Thus, only a small fraction of the output monochromator or the research sample signals is detected by the spectrometer.

We hypothesize that using a larger spectrometer slit or a larger core fiber will yield better results in terms of signal intensity, which, however, will be at the expense of reduced spectral resolution. The complexity of the fluorescent peaks of different fluorophores or distinctive peaks of a single fluorophore in a food matrix may merge and no longer be measured separately. For these reasons, an optical fiber spectrometer with interchangeable slits will be a more flexible solution for EEM spectroscopy in food system analysis. In principle, it is possible to combine the radiation from the LED with the induced fluorescence. In this way, the useful width of the light spectrum will increase, allowing for the broader tuning of the radiation from the monochromator.

## 5. Conclusions

To the best of our knowledge, for the first time in the scientific literature, we demonstrate the possibility of using LED-induced fluorescence from an organic dye as a light source with potential applications in EEM fluorescence spectroscopy for food system analysis. The experimental results show that fluorescence can be induced and a detectable signal can be obtained from both fluorophores with a smooth spectral profile like Coumarin 6 or those with a specific profile like Perylene. It was demonstrated that direct excitation, without an optical fiber, was more suitable when using LEDs at which a sufficiently intense fluorescent signal is generated. The ability to connect the output of the monochromator to an optical fiber allowed for the testing of samples with a volume of the order of 1 μL.

## Data Availability

The original contributions presented in the study are included in the article, further inquiries can be directed to the corresponding author.
